# Five Advances in the Last 50 Years That Have Impacted Endocrine Surgery

**DOI:** 10.1002/wjs.70305

**Published:** 2026-04-11

**Authors:** Matilda Anneback, Priya H. Dedhia, Aimee Di Marco, Els Nieveen van Dijkum, Julie A. Miller, Janice L. Pasieka

**Affiliations:** ^1^ Department of Surgical Sciences Uppsala University Hospital Uppsala Sweden; ^2^ Division of Surgical Oncology Department of Surgery The Ohio State University Comprehensive Cancer Center and Wexner Medical Center Columbus Ohio USA; ^3^ Department of Endocrine Surgery Imperial College Healthcare NHS Trust Hammersmith Hospital London London UK; ^4^ Amsterdam UMC Gastroenterology Endocrinology Metabolism Cancer Center Amsterdam the Netherlands; ^5^ The Royal Melbourne Hospital Melbourne Victoria Australia; ^6^ Cumming School of Medicine University of Calgary Calgary Alberta Canada

**Keywords:** adrenal, endocrine surgery, neuroendocrine tumors, parathyroid, thyroid

Fifty years ago, the nidus for forming a focused group of international surgeons dedicated to the field of endocrine surgical diseases started to evolve. At the 1979 Société Internationale de Chirurgie congress in San Francisco, a gathering of global thought‐leaders held the inaugural meeting of International Association of Endocrine Surgeons (IAES). The proceedings of that first meeting, which were published 45 years ago in this journal, catalyzed the evolution of endocrine surgery as we know it today [1, 2, 3, 4, 5, 6, 7, 8, 9]. Since then, IAES members have driven world‐wide collaboration and innovation in hereditary and sporadic endocrine surgical diseases.

The past five decades have witnessed improvements in biochemical testing, allowing for better diagnostic accuracy in adrenal diseases and moved primary hyperparathyroidism (pHPT) from a clinical diagnosis to a biochemical one allowing for intervention before significant end‐organ damage. Fine needle aspiration (FNA) developed and honed by our Swedish colleagues has revolutionized our ability to diagnose thyroid cancer preoperatively [2]. Advances in bedside ultrasound (US) and cross‐sectional imaging have changed the landscape for surgical planning in all endocrine surgical diseases [3, 10]. Surgical innovations such as endoscopic approaches to the adrenal, pancreas, and thyroid have drastically changed postoperative care and patient outcomes [11, 12, 13]. What started as a revolutionary palliative drug, for patients with carcinoid syndrome, the development of the somatostatin analog octreotide, led to the development of functional imaging modalities and theranostic treatments for metastatic neuroendocrine tumors (NETs) [14]. From this landscape of progress, we highlight five transformative advances: genetic discoveries in MEN syndromes, refinements in adrenal diagnostics and surgical intervention, the de‐escalation revolution in thyroid surgery, the diagnostic evolution of pHPT, and the development of theranostics for NETs. The most revolutionary among these has been our understanding of genetic mutations in syndromic endocrine diseases, which reshaped not only endocrine surgery but all of oncology [15, 16].

## 1 From Genetic Discovery to Surgical Strategy

The discovery of the *MEN1* and *RET* genes represents one of the most influential advances in endocrine surgery over the past 50 years. Their association with multiple endocrine neoplasia type 1 (MEN1) and type 2 (MEN2) syndromes provided a paradigm for how genetic insight can reshape surgical screening, timing, and preventive intervention. By enabling presymptomatic identification of mutation carriers and structured surveillance with prophylactic surgical strategies, these discoveries shifted the focus from treatment of manifest disease to proactive management of populations at risk.

MEN1 was recognized clinically long before the molecular era as an autosomal dominant hereditary disorder involving the parathyroid glands, anterior pituitary, and duodenopancreatic NETs [17]. Genetic mapping in the late 1980s localized the MEN1 locus to chromosome 11q13, and positional cloning in 1997 identified MEN1, encoding the tumor suppressor protein menin [18, 19]. These discoveries explained the multicentric and asynchronous tumor development characteristic of MEN1 and clarified why limited resections frequently failed. Surgical management therefore evolved toward standardized multiglandular parathyroid surgery—most commonly subtotal parathyroidectomy—aimed at durable biochemical control. For pancreatic NETs, genetic insight supported risk‐adapted strategies ranging from surveillance and parenchyma‐sparing procedures, such as enucleation, to formal pancreatic resection based on tumor size and biological behavior [20]. These strategies are underpinned by cascade screening of families and lifelong surveillance across multiple organs.

A more direct translation of genetic discovery into surgical timing is seen in MEN2. MEN2 is characterized by medullary thyroid carcinoma, often presenting early in life and frequently accompanied by pheochromocytoma and, in some variants, pHPT. In 1993, activating germline mutations in the *RET* proto‐oncogene were independently identified as the genetic basis of MEN2 [21, 22]; codon–phenotype correlations emerged shortly thereafter and now guide surgical timing and management. These correlations were subsequently incorporated into international guidelines that recommend mutation‐based timing of prophylactic thyroidectomy and screening, directly linking genetic risk stratification to surgical decision‐making [23].

These advances illustrate how genetic discovery has transformed endocrine surgery from reactive treatment to anticipatory strategy‐driven care for patients with hereditary endocrine disease [15, 16].

## 2 The Who, When, and How of Adrenalectomy

Care of patients with surgical adrenal disease has advanced remarkably in the last half century. Biochemical assessment and high‐resolution cross‐sectional imaging have enabled surgeons to provide sound advice and plan safe procedures. In addition, minimally invasive techniques have transformed surgical outcomes.

The introduction of computed tomography (CT) in the 1970s enabled reliable detection of adrenal masses, replacing exploratory surgery with accurate noninvasive localization [24]. Modern multidetector CT provides high‐resolution imaging that often distinguishes benign lipid‐rich adenomas from malignant or metastatic disease [10]. Magnetic resonance imaging (MRI) and functional scintigraphy are also valuable in select cases [10, 25]. Accurate reliable imaging has dramatically enhanced preoperative planning by defining tumor size and margins, vascular relationships, and bilateral or nodal disease. It has also revealed a large volume of incidental adrenal masses, enhancing our understanding of prevalence and natural history of adrenal masses [26].

Progress in adrenal biochemical testing has been equally transformative. Sensitive immunoassays and mass‐spectrometry allow evaluation of cortisol, aldosterone, and catecholamine excess, distinguishing nonfunctioning tumors from those with autonomous secretion. The first serum cortisol radioimmunoassay (RIA) developed in the early 1970's evolved into automated immunoassays in the 1990's, allowing use of the dexamethasone suppression test to screen for autonomous cortisol production [27]. RIA methods for aldosterone and renin became generally available in the 1980's, and plasma metanephrines only since the 1990's [28, 29].

Although imaging and biochemical investigations have refined patient selection for surgery, minimally invasive surgical techniques introduced and refined by IAES members have redefined adrenalectomy as a safe procedure with exceedingly low morbidity, short hospital stays, and rapid recovery. Laparoscopic adrenalectomy, introduced by Gagner in 1992, rapidly became gold standard for most benign adrenal tumors, offering reduced postoperative pain, shorter hospital stays, and faster recovery compared with open procedures [12]. The posterior retroperitoneoscopic approach popularized by Walz in the early 2000's further minimized dissection while improving visualization and access, particularly in patients with prior abdominal surgery [13].

These innovations have fundamentally reshaped adrenal surgery: patients are diagnosed earlier, selected more appropriately for operative management, and treated with safer less invasive techniques, leading to markedly improved clinical outcomes.

## 3 The De‐Escalation Revolution in Thyroid Surgery

Over the past five decades, thyroid surgery has evolved toward increasingly conservative management. Evidence now shows that less invasive approaches can often achieve equivalent—or even improved—patient‐centered outcomes with fewer complications. This evolution began in the 1980s, when the widespread adoption of fine‐needle aspiration (FNA) fundamentally changed evaluation of thyroid nodules. Our past‐president P ‐O Granberg and pathologist T. Lowhagen, demonstrated FNA's high diagnostic accuracy, which enabled surgeons to distinguish benign from malignant disease preoperatively, leading to the development of standardized thyroid cytology: the Bethesda System [2, 30, 31]. As FNA became standard of care worldwide, surgical intervention rates dropped by nearly 50%, sparing countless patients from diagnostic thyroidectomies [32].

Beyond diagnosis, the management of benign thyroid disease underwent similar de‐escalation. Once common practices, such as suppressive levothyroxine therapy for euthyroid benign nodules, have now been abandoned after trials demonstrated only modest benefits while risking iatrogenic hyperthyroidism [33]. For benign nodules requiring surgery, the approach shifted from total thyroidectomy (TTx) to lobectomy when feasible, preserving thyroid function and avoiding lifelong hormone dependence [34].

In the cancer realm, pioneering surgeons such as Blake Cady challenged the prevailing dogma of TTx, advocating instead for lobectomy in low‐risk differentiated thyroid cancer [4, 35]. With accumulating evidence of excellent outcomes for conservative surgery, the American Thyroid Association endorsed lobectomy as preferred therapy for low‐risk tumors ≤ 4 cm [36]. De‐escalation continued beyond surgery itself: selective use of radioactive iodine spared thousands of low‐risk patients from adjuvant therapy [37].

Perhaps, the most dramatic shift emerged with active surveillance for papillary microcarcinoma. Beginning in 1993, Miyauchi's pioneering cohort at Kuma Hospital demonstrated that observation of low‐risk papillary microcarcinoma ≤ 1 cm (mPTC) produced oncologic outcomes equivalent to immediate surgery—with only 8% showing tumor enlargement and 4% developing nodal metastasis at 10 years, all successfully salvaged with delayed surgery [38, 39]. In his 2015 IAES presidential address, Miyauchi reported that immediate surgery resulted in 6‐ to 20‐fold higher complication rates of vocal cord paralysis and hypoparathyroidism cementing the global acceptance of observation for low‐risk mPTC [40]. The demonstration of the indolent nature these small PTC opened the door for other investigators to the move away from routine TTx in low‐risk PTC [36].

Looking forward, molecular testing of indeterminate nodules promises to further refine patient selection and avoid unnecessary diagnostic surgery as decreasing costs improve widespread availability [41]. This journey of progressive de‐escalation, from routine TTx to active surveillance demonstrates how evidence‐based practice transforms surgical care, prioritizing patient quality of life and safety without compromising cure, a philosophy the IAES community has championed for decades.

## 4 Hyperparathyroidism: From Clinical to Biochemical Diagnosis

The past 50 years has seen an increase in the prevalence of pHPT beyond that caused by the introduction of routine serum calcium estimation in the 1970's [42]. This increase can be accounted for by changes in clinical practice which have opened reservoirs of pHPT: (1) incidental diagnosis with absent or subclinical end organ changes, (2) post‐menopausal females subjected to routine bone density screening, (3) biochemically mild pHPT in whom the diagnosis is made with increased confidence due to high quality data on 24‐h urinary calcium and access to genetic testing excluding familial hypercalcemia hypocalciuric (FHH), and (4) normocalcaemic and normohormonal pHPT which have been recognized as relevant clinical entities. International consensus guidelines for standard pHPT were first developed in 1990 and have since been re‐developed; however, specific guidance on normocalcaemic HPT is still lacking [43].

Medical therapy with calcimemetic drugs was first described in 2003 [44] but subsequently recognized as inferior to parathyroidectomy in treating end organ consequences in both renal and primary disease. Parathyroidectomy therefore remains the only definitive treatment.

Despite early studies presented at IAES meetings on the utilization of US in pHPT [3, 45], in 1986, the radiologist John Doppman remarked that “the only localizing study indicated in a patient with untreated primary hyperparathyroidism is to localize an experienced parathyroid surgeon” reflecting the lack of reliable localization studies and reliance on bilateral neck exploration. Since then, improvements in localization studies have abounded, with many IAES surgeons collaborating across disciplines. Technological advances in US with high frequency linear transducers and the use of Doppler, alongside experience and specialization of radiologists and/or surgeon‐directed US increased the yield even for small adenomas. The technique of 4DCT was described by IAES members from MD Anderson in 2006 [46] and exploded in use due to ease of access [47]. However, it is nuclear medicine which stole the show through functional imaging, initially in the late 1980s with the tracer 99mTc‐sestamibi [48] and then 11C‐Choline and 18F‐Fluoro Choline in 2014 [49, 50, 51]. These advances permitted the development of minimally invasive parathyroid surgery, targeting a single gland and offering selected patients the opportunity for cure through a smaller incision, and rendered interventional imaging prior to re‐operative parathyroidectomy redundant.

Intraoperative adjuncts have also been developed: the first rapid parathyroid hormone (PTH) assay with turnaround time of under 10 min to confirm cure was described in 1991 by George Irwin [52] and more recently, autofluorescent imaging to identify parathyroid tissue [53]. However, bilateral neck exploration remains the only treatment for multiglandular disease, whether it is recognized as such pre‐operatively, with data showing equivalent cure rates in large centers irrespective of imaging [54] or intraoperative PTH use [55].

Besides diagnostics, the greatest impact on patient outcomes has likely been through training and specialization of parathyroid surgeons with data on outcomes for parathyroidectomy by high volume surgeons testament to this and John Doppman's advice [56].

## 5 Theranostics in NET Started Only 40 years ago

Theranostics, combining diagnostic imaging and therapy in one substance, dates to 1941 when radioactive iodine was used for patients with hyperthyroidism and thyroid cancer [57, 58]. Theranostics for NETs was first discussed at a Rotterdam meeting in 1985, by Steven Lamberts, Jean Claude Reubi and Larry Kvols when receptors of somatostatin were identified on neuroendocrine cells [59], The discussion was then started on combining the somatostatin analog, octreotide, synthesized in 1982 [60] with radiolabeling for imaging and therapeutics [61, 62].

The imaging of NETs with radiolabeled octreotide was studied and improved by the Swedish team in Uppsala, led by IAES members Goran Akerstrom and Kjell Öberg [63]. The focus of this team was initially the diagnostic part of labeling octreotide, whereas the Rotterdam team, with Eric Krenning as catalyst, was searching for therapeutic options. This led to the first patient with a glucagonoma treated in 1992 with high doses of ^111^IN‐pentetreotide [64]. From that moment, with the start of octreotide‐based peptide receptor radionuclide therapy (PRRT) for NETs in 1992, many centers embraced and improved the PRRT concept [65, 66]. Led by our European colleagues, clinical phase I and II trials demonstrated biochemical and clinical responses and improvements in progression free survival (PFS), with acceptable toxicity [67, 68]. New labeling, kidney protection, shorter intervals, and combination therapy of PRRT with systemic treatment have further advanced its utilization. The marked improvement in PFS seen in NETTER‐1 phase III trial in 2017, led to the global utilization of PRRT for progressive NETs [69]. Surgeons have since started to use PRRT as a neo‐adjuvant option, although large trials are lacking some evidence supports this treatment [70].

Current treatment of NETs has evolved only in the recent 4 decades from debulking surgery as palliative option for extreme hormonal overproduction to survival improvement with sensitive diagnostics and specific treatment based on radiolabel octreotide [14].

Medicine and surgery have evolved drastically over the last 50 years, in part due to advances in science and technology. However, without global collaboration across disciplines, knowledge transfer through peer‐review publications, fellowship training, scientific meetings, and collegial networking we would likely not have witnessed the marked improvement in patient care and outcomes in such a short period of time.

## Author Contributions

**Matilda Anneback:** conceptualization, writing – original draft, writing – review and editing. **Priya H. Dedhia:** conceptualization, writing – original draft, writing – review and editing. **Aimee Di Marco:** conceptualization, writing – original draft, writing – review and editing. **Els Nieveen van Dijkum:** conceptualization, writing – original draft, writing – review and editing. **Julie A. Miller:** conceptualization, writing – original draft, writing – review and editing. **Janice L. Pasieka:** conceptualization, writing – original draft, writing – review and editing.

## Funding

The authors have nothing to report.

## Conflicts of Interest

The authors declare no conflicts of interest.

## Data Availability

Data sharing not applicable to this article as no datasets were generated or analysed during the current study.
